# Associations of pubertal stage and body mass index with cardiometabolic risk in Hong Kong Chinese children: A cross-sectional study

**DOI:** 10.1186/s12887-015-0446-0

**Published:** 2015-09-24

**Authors:** Noel PT Chan, Kai C Choi, E Anthony S Nelson, Juliana C Chan, Alice PS Kong

**Affiliations:** The School of Nursing, The University of Hong Kong, 4/F, William M.W. Mong Block, 21 Sassoon Road, Pokfulam, Hong Kong SAR, China; The Nethersole School of Nursing, The Chinese University of Hong Kong, 7th floor, Esther Lee Building, Shatin, N.T., Hong Kong SAR, China; Department of Paediatrics, The Chinese University of Hong Kong, Hong Kong SAR, China; Department of Medicine and Therapeutics, The Chinese University of Hong Kong, Hong Kong SAR, China

**Keywords:** Pubertal stage, Body mass index, Cardiometabolic risk, Childhood overweight/obesity, Waist circumference

## Abstract

**Background:**

Puberty is associated with a clustering of cardiometabolic risk factors (CMRFs) during adolescence that are manifested in later life. Although anthropometric variables such as body mass index (BMI) can predict cardiometabolic risk in children and adolescents, it is not clear whether there is an interaction between pubertal stage and BMI associated with cardiometabolic risk in this age group. This paper examines the association of pubertal stage and BMI with CMRFs in Hong Kong Chinese children.

**Methods:**

A cross-sectional school-based study was conducted among 1985 (95.1 %) students aged 6 to 18 years. Fasting lipid profile and plasma glucose, blood pressure, body weight, body height and waist circumference were measured. A self-reported pubertal stage questionnaire was used to assess pubertal stage of participants**.** Two cardiometabolic risk scores, alpha and beta, were constructed to quantify cardiometabolic risk. Cardiometabolic risk score alpha refers to the sum of z-scores of sex-specific, age-adjusted waist circumference, height-adjusted systolic and diastolic blood pressure, fasting plasma glucose, triglyceride and low-density lipoprotein cholesterol, and minus z-score of sex-specific age-adjusted high-density lipoprotein cholesterol. Cardiometabolic risk score beta includes all components of risk score alpha except waist circumference.

**Results:**

The interaction of BMI z-score (ZBMI) and pubertal stage demonstrated a further increase in variance explained in both the cardiometabolic risk scores alpha and beta (0.5 % and 0.8 % respectively) in boys and (0.7 % and 0.5 % respectively) in girls.

**Conclusions:**

Pubertal stage has an interaction effect on the association of cardiometabolic risk by BMI in boys and may have a similar but lesser effect in girls.

## Background

Puberty is a critical period of growth and development and is associated with dramatic changes of hormonal and body composition [1]. Accumulating evidence suggests that puberty is associated with a clustering of cardiometabolic risk factors (CMRFs) in later life [2–6]. Early onset of puberty has been associated with higher adult body mass index (BMI), fasting insulin, diastolic blood pressure (DBP), and decreased high-density lipoprotein cholesterol (HDL-C) in both sexes and with higher total serum cholesterol (TC), low-density lipoprotein cholesterol (LDL-C), and triglyceride (TG) in males [7]. Early menarche has been associated with an increased risk of type 2 diabetes in adulthood even when controlled for adult BMI [2]. A longitudinal study in adolescent girls found that early menarche was associated with increased cardiovascular risk including elevated blood pressure and glucose intolerance compared with later maturing girls, but independent of age, free fat mass and percent body fat [5]. Early sexual maturation has also been positively associated with increased BMI and skinfold thickness in girls, whereas boys have a reverse association and were also thinner when compared to girls [8]. Consistent with previous findings [8–10], a multicenter longitudinal study found that boys with a higher BMI were more likely to be classified as late maturers [11]. All these studies indicate that pubertal stage may have an association with cardiometabolic risk. In parallel, a growing body of evidence indicates that BMI of children and adolescents can predict their cardiometabolic risk [12, 13]. However, there is a lack of research into the possible interaction effect of pubertal stage on the association of cardiometabolic risks by BMI. The current study aimed to explore the interaction effect of pubertal stage on the association of cardiometabolic risks by BMI in Hong Kong Chinese children.

## Methods

### Participants and setting

This study was a sub-study of a large, school-based, cross-sectional study funded by the Hong Kong Research Grants Council (CUHK4465/06 M) and was conducted between 2007 and 2008 [14]. A complete list of all primary and secondary schools of all 18 districts was obtained from the Education Bureau of Hong Kong to compile a sampling frame of all local schools. A two-stage cluster sampling method was employed. In the first stage of the sampling, one primary school and one secondary school were randomly selected from each of all districts in Hong Kong using a computer-generated coding system. Among all schools, five primary and six secondary schools were randomly selected and enrolled in the study with support of school principals. In stage two, two classes in each grade were selected in collaboration with the school principal. All Hong Kong Chinese students of the selected classes were invited to join the study [14].

A total of 2119 participants aged 6–20 years, 804 primary and 1315 secondary school students, were recruited. Of these, 31 participants were excluded owing to active medical/psychiatric illnesses or use of long term medications (*n* = 17) and aged 19 or above (*n* = 14). Among the 2088 (99.3 %) eligible participants aged 6–18 years, 1985 (95.1 %) had completed a self-reported Tanner pubertal questionnaire and were eligible to enter data analysis. Ethical approval was obtained from the Clinical Research Ethics Committee of the Chinese University of Hong Kong. Informed assent was obtained from all participants together with their parents’ informed written consent before they were entered into the study.

All data collection, including anthropometric measurements and blood taking procedures, were completed in the schools between 07:30 and 08:30 before their first school lesson as fasting blood samples were required. Although parents were told that they could accompany their children during the data collection, the majority of children had their blood taken and other data collection procedures undertaken without the presence of their parents.

#### Procedure

The students were given a self-administrated questionnaire to take home for completion. Data collected included demographic information, pubertal staging [15], history of medical/psychiatric illness, and use of any long term medications. Secondary school students were asked to complete the questionnaire by themselves and primary school students were asked to seek help from their parents/guardians. Children were instructed to return the questionnaire on the day of the survey and to fast overnight for at least 8 h.

#### Data collection

The children’s body weight (BW), body height (BH) and waist circumference (WC) were measured by trained research staff. BW was measured to 0.1 kg on a calibrated weight scale (Tanita physician digital scale, model number TBF-410, Tanita Corp., Tokyo, Japan) with children standing without shoes, lightly clothed. A correction of 0.5 kg was made for clothing for all children. Standing BH was measured to the nearest 0.1 cm using a portable rigid stadiometer. WC was measured twice to the nearest 0.1 cm. The WC measurement site was located midway between the lowest rib and the superior border of the iliac crest at the mid-axillary line on bare skin during expiration, while standing straight-up using a non-stretchable flexible measuring tape. The two measurements were then averaged for data analysis. The children’s blood pressure (BP) was measured twice from the right arm after at least 5 min of rest in a seated relaxed position by a validated electronic BP monitor (Omron T5, Omron Healthcare Inc., Tokyo, Japan). The BP values were the average of the two readings. The time interval for data collection between self-reported and measured anthropometric values was less than 2 weeks.

#### Collection of blood samples of the metabolic profile

Fasting blood samples were collected for the measurement of plasma glucose and lipid profile including TC, TG, LDL-C and HDL-C levels. All blood samples were kept in ice at 0 °C and returned to the laboratory within 4 h after collection either for assay or storage. Blood samples including fasting plasma glucose (FPG) and lipid profile were assayed within 6 h after collection and additional aliquots of serum for other assays were stored at −70 °C. Glucose (hexokinase method), TC (enzymatic method), TG (enzymatic method without glycerol blanking) and HDL-C (direct method using PEG-modified enzymes and dextran sulfate) were measured on a Roche Modular Analytics system (Roche Diagnostics GmbH, Mannheim, Germany) using standard reagent kits supplied by the manufacturer of the analyzer. The precision performance of these assays was within the manufacturer's specifications.

#### Definitions of cardiometabolic risk factors (CMRFs)

We adopted the definition from Cruz and colleagues [16] to define clustering of CMRFs. All cutoff values were based on data from local school children [12, 13, 17]. Specifically, children who had three or more out of the following five CMRFs were considered as having a clustering of CMRFs:

i. TG ≥90th percentile (age- and sex-specific);
ii. FPG ≥5.6 mmol/L;
iii. fasting HDL-C ≤10th percentile (age- and sex-specific);
iv. WC ≥90th percentile (age- and sex-specific);
v. either systolic blood pressure (SBP) or diastolic blood pressure (DBP) ≥90th percentile (age, sex and height specific).

#### The definition of pre-pubertal, pubertal and late/post-pubertal stage

The self-reported Tanner pubertal questionnaire was used for data collection [18]. The scores of the two items of each of the 5 Tanner pubertal stages in each sex [19] (female breast, male genitalia development and pubic hair growth in both sexes) were averaged and rounded up to the highest pubertal composite stage so as to avoid underestimating the pubertal stage [15]. The roundup of the 5 composite pubertal stages were then re-classified into 3 pubertal stages: pre-pubertal stage (equivalent to the Tanner pubertal stage 1), pubertal stage (average of Tanner pubertal stages 2 and 3), and late/post-pubertal stage (average of Tanner pubertal stages 4 and 5).

### Statistical analyses

Data were summarized and presented by appropriate descriptive statistics. Continuous and categorical data were presented as mean (standard deviation) and frequency (%), respectively, for illustrating the sample characteristics. TG values were logarithmically transformed to correct for skewness before being subjected to analysis. BMI was calculated as BW in kilograms divided by BH in meters squared (kg/m^2^). Chi-square test and one-way ANOVA were used to examine the association between pubertal stage and CMRFs. Despite the hierarchical nature of the data, students recruited from the same class/school/district (cluster) are unlikely correlated with one another with respect to the outcome variables (cardiometabolic risk factors) since they are all individual physiologically based measures. In this regard, variation between clusters would be ignorable as compared to variation between individual students. Thus the analysis of the study was conducted on the basis of a single-level model accounting for variations between individuals only. All the statistical analyses were performed using IBM SPSS 22.0 (IBM Crop., Armonk, NY, USA). All statistical tests were two-sided and a *p*-value <0.05 was considered statistically significant.

#### The effect of pubertal stage on the association of CMRFs by BMI

In view of the relatively small number of children (*n* = 54) having a clustering of CMRFs, a summary risk score, based on The European Youth Heart Study [20] was constructed to quantify cardiometabolic risk for the population sample of school children in Hong Kong. The components of the score were selected on the basis of the International Diabetes Federation [21] and the modified National Health and Nutrition Survey [13] definitions of metabolic syndrome. The risk score **α** was computed by summing up the following: z-score of sex-specific age-adjusted WC, TG, LDL-C, FPG, minus z-score of sex-specific age-adjusted HDL-C, and the greater one of the two z-scores with sex-specific age and height-adjusted SBP and DBP.

Each of the component variables of the risk score was regressed with age (and with BH for SBP and DBP) for boys and girls separately. The standardized residuals were retained to represent the z-score of age-adjusted values for each of the component variables. In parallel, a CMRF score β without the central obesity component (i.e. WC) was also calculated for comparison. Hierarchical multiple regression analyses were used to examine the interaction effect of pubertal stage on the association of CMRFs by BMI. All the statistical analyses were conducted separately for boys and girls. Z-score of unadjusted BMI (ZBMI) was first entered into regression model with the CMRFs score as the dependent variable. Then pubertal stage was recoded as two dummy variables (pubertal and post-pubertal with pre-pubertal as reference) and entered into the regression. Finally, the interaction terms of the pubertal stages and ZBMI were entered into the regression model. The significance of the additional included terms as compared with the preceding model was assessed using the F-test. The interaction effect of pubertal stage was indicated by the significance of the interaction terms added to the regression model.

#### Power analysis

A sample size of 828 boys and 1157 girls would allow a regression analysis of BMI and pubertal stage to detect an interaction effect as small as R^2^ = 0.01, R^2^ = 0.008 and R^2^ = 0.006 with, respectively, 86 %, 78 % and 65 % power for boys, and 95 %, 90 % and 80 % power for girls, respectively, at 5 % level of significance, given that the main effects of BMI and pubertal stage have already accounted for 10 % variance of the cardiometabolic risk score.

## Results

Of 2088 children aged 6 to 18 years, 1985 (95.1 %) children completed the self-reported Tanner pubertal questionnaire. The clinical characteristics of the children who did and did not complete the self-reported Tanner pubertal questionnaire were similar. The demographic and clinical characteristics, including Tanner pubertal stages, of boys and girls are illustrated in Table 1.

**Table 1:** Demographic and clinical characteristics of study cohort

Characteristics	Male (*n* = 828)	Female (*n* = 1157)
Age (years)	12.9 (3.2)	13.6 (3.3)
Weight (kg)	46.6 (14.9)	43.5 (11.7)
Height (cm)	154.3 (17.1)	150.9 (13.0)
Body Mass Index (kg/m^2^)	19.1 (3.5)	18.7 (3.1)
Waist circumference (cm)	66.5 (9.9)	63.9 (7.9)
Systolic blood pressure (mmHg)	113.6 (12.1)	107.1 (9.9)
Diastolic blood pressure (mmHg)	66.7 (8.7)	66.8 (8.0)
HDL-C (mmol/L)	1.6 (0.3)	1.6 (0.3)
LDL-C (mmol/L)	2.1 (0.6)	2.2 (0.6)
Triglyceride (mmol/L)^**†**^	0.7 (0.6 – 1.0)	0.7 (0.6 – 1.0)
Fasting plasma glucose (mmol/L)	4.8 (0.4)	4.7 (0.3)
Obesity status^ψ^		
Normal	623 (75.2 %)	968 (83.7 %)
Overweight	129 (15.6 %)	128 (11.1 %)
Obese	76 (9.2 %)	61 (5.3 %)
Puberty (Tanner stage)^ψ^		
1	205 (24.8 %)	178 (15.4 %)
2	177 (21.4 %)	151 (13.1 %)
3	129 (15.6 %)	219 (18.9 %)
4	284 (34.3 %)	464 (40.1 %)
5	33 (4.0 %)	145 (12.5 %)

The cohort consisted of children aged 6–18 years who completed the pubertal assessment questionnaire

Data marked with^†^ were presented as medians (interquartile ranges)

Data marked with^ψ^ as frequencies (%), all others were presented as means (SD)

Overweight: Body mass index (BMI) greater than or equal to 85th percentile and <95th percentile (age- and sex-specific)

Obese : BMI ≥ 95^th^ percentile (age- and sex-specific)

HDL-C: High density lipoprotein cholesterol); LDL-C (Low density lipoprotein cholesterol)

### Association between pubertal stage and CMRFs

For boys, puberty was significantly associated with several CMRFs, including increased WC (*p* = 0.007), high TG (*p* = 0.011) and high BP (*p* = 0.001). Puberty was also significantly associated with overweight (*p* <0.001) and obesity (*p* <0.001) (Table 2). The highest rates for boys of increased WC (23.4 %), high BP (28.3 %), high TG (14.4 %), high CMRFs clustering (4.6 %), overweight (37.6 %) and obesity (18 %) were all found in the pre-pubertal group. For girls, pubertal stage was significantly associated with increased WC (19.7 %, *p* = 0.005) in the post-pubertal group and high BP (18 %, *p* = 0.033) in the pre-pubertal group (Table 2).

**Table 2:** Association between pubertal stage and cardiometabolic risk factors (CMRFs)

	Male (*n* = 828)				Female (*n* = 1157)			
	Pre-pubertal^1^ (*n* = 205)	Pubertal^2^ (*n* = 306)	Late /Post-pubertal ^3^ (*n* = 317)	*p*-value^4^	Pre-pubertal^1^ (*n* = 178)	Pubertal^2^ (*n* = 370)	Late/Post-pubertal^3^ (*n* = 609)	*p*-value ^4^
Age (y) [median (IQR)]	9.5 (8.3 – 11.0)	12.1 (10.5 – 13.5)	16.0 (15.1 – 17.2)		8.4 (7.6 – 9.3)	12.1 (10.8 – 13.8)	15.9 (14.7 – 17.3)	
Cardiometabolic risk factors [*N*(%)]								
Increased waist circumference^5^	48 (23.4 %)	59 (19.3 %)	41 (12.9 %)	0.007	18 (10.1 %)	55 (14.9 %)	120 (19.7 %)	0.005
High triglyceride^6^	28 (14.4 %)	29 (9.7 %)	20 (6.3 %)	0.011	15 (9.0 %)	46 (12.5 %)	55 (9.0 %)	0.190
Low HDL-C^7^	18 (9.2 %)	28 (9.3 %)	26 (8.2 %)	0.873	17 (10.2 %)	26 (7.1 %)	35 (5.7 %)	0.128
High fasting plasma glucose^8^	3 (1.5 %)	7 (2.3 %)	10 (3.2 %)	0.573	0 (0.0 %)	9 (2.4 %)	7 (1.1 %)	0.065
High blood pressure^9^	58 (28.3 %)	62 (20.3 %)	46 (14.5 %)	0.001	32 (18.0 %)	62 (16.8 %)	72 (11.8 %)	0.033
Clustering of the above CMRFs^10^	9 (4.6 %)	12 (4.0 %)	10 (3.2 %)	0.705	3 (1.8 %)	10 (2.7 %)	10 (1.6 %)	0.495
Overweight/obese^11^	77 (37.6 %)	79 (25.8 %)	49 (15.5 %)	<0.001	28 (15.7 %)	57 (15.4 %)	104 (17.1 %)	0.768
Obese ^12^	37 (18.0 %)	30 (9.8 %)	9 (2.8 %)	<0.001	9 (5.1 %)	21 (5.7 %)	31 (5.1 %)	0.915
Cardiometabolic Risk Score [mean (SD)]								
Cardiometabolic Risk Score α^13^	0.65 (3.38)	0.38 (3.26)	0.55 (3.05)	0.620	−0.49 (2.96)	0.22 (2.72)	0.41 (2.92)	0.002
Cardiometabolic Risk Score β^14^	0.61 (2.88)	0.36 (2.60)	0.60 (2.59)	0.460	−0.08 (2.45)	0.23 (2.30)	0.28 (2.46)	0.220

Pre-pubertal :Tanner pubertal stage 1

Pubertal : Tanner pubertal stages 2 and 3

Late/Post-pubertal : Tanner pubertal stages 4 and 5

*p-value testing the statistical significance of the association between each of the CMRFs and pubertal stage

Waist circumference ≥90^th^ percentile (age- and sex-specific)

Triglyceride ≥90^th^ percentile (age- and sex-specific)

High density lipoprotein cholesterol (HDL-C) ≥10th percentile (age- and sex-specific)

Fasting plasma glucose ≥ 5.6 mmol/L

Systolic blood pressure (BP)/diastolic BP ≥90th percentile (age, sex and height specific)

^†^Clustering of cardiometabolic risk factors (CMRFs) was defined as 3 or more of the above 5 CMRF

Overweight : Body mass index (BMI) ≥85th percentile and <95th percentile (age- and sex-specific)

Obese : BMI ≥95^th^ percentile (age- and sex-specific)

Cardiometabolic risk score α = Sum of components’ z score: Components of cardiometabolic risk score α includes z-score of sex-specific, age-adjusted waist circumference, systolic and diastolic blood pressure (also height-adjusted), fasting plasma glucose, triglyceride and low-density lipoprotein cholesterol (LDL-C), and minus z-score of sex-specific age-adjusted high-density lipoprotein cholesterol (HDL-C). Cardiometabolic risk score β includes all components of risk score α except waist circumference

### Cardiometabolic summary risk scores: α and β

Summary risk scores, **α** and **β**, to quantify the cardiometabolic risk were used to examine the interaction effect of pubertal stages on the association of cardiometabolic risk by BMI in the analyses. The variables used for assessing the interaction effect of pubertal stage on the association of cardiometabolic risk using a summary risk score **α** in the hierarchical regression analyses were ZBMI, pubertal stage and interaction of ZBMI and pubertal stage. In Model 1, ZBMI explained a significant proportion of the variance [R^2^(95 % CI)] in cardiometabolic risk score **α** in both boys [35.0 %,(29.7 % - 40.3 %)] and girls [22.3 %,(18.1 % - 26.6 %)]. Pubertal stage was entered in Model 2 and accounted for a significant increase in variance explained in both sexes, R^2^ = 37.4 % (32.1 % - 42.7 %) in boys and R^2^ = 26.1 % (21.7 % - 30.5 %) in girls. When the interaction-term of ZBMI and puberty was entered in Model 3, there was a further significant increase of the variance explained in both sexes, R^2^ = 37.9 % (32.7 % - 43.1 %) in boys and R^2^ = 26.8 % (22.4 % - 31.2 %) in girls (Table 3a).

**Table 3a:** Effect of pubertal stage interaction on association of cardiometabolic risk score α by BMI

Cardiometabolic Risk Score α *	Boys (*n* = 828)					Girls (*n* = 1157)				
	B	SE	*P*	R^2^ (95 % CI)	*p* (△F)	B	SE	*p*	R^2^ (95 % CI)	*p* (△F)
Model 1				35.0 % (29.7 % – 40.3 %)	<0.001^#^				22.3 % (18.1 % – 26.6 %)	<0.001^#^
ZBMI	1.894	0.091	<0.001			1.364	0.075	<0.001		
Model 2				37.4 % (32.1 % – 42.7 %)	<0.001				26.1 % (21.7 % – 30.5 %)	<0.001
ZBMI	2.019	0.092	<0.001			1.722	0.088	<0.001		
Pubertal stage:										
Pre-pubertal (ref)										
Pubertal	−0.594	0.235	0.012			−0.460	0.240	0.055		
Late/Post-pubertal	−1.302	0.238	<0.001			−1.622	0.253	<0.001		
Model 3				37.9 % (32.7 % – 43.1 %)	0.024				26.8 % (22.4 % – 31.2 %)	0.006
ZBMI	1.747	0.164	<0.001			2.485	0.255	<0.001		
Pubertal stage:										
Pre-pubertal (ref)										
Pubertal	−0.477	0.239	0.046			−1.260	0.346	<0.001		
Late/Post-pubertal	−1.196	0.241	<0.001			−2.340	0.338	<0.001		
Interaction terms:										
ZBMI * Pubertal	0.593	0.224	0.008			−0.903	0.302	0.003		
ZBMI * Late/Post-pubertal	0.177	0.230	0.443			−0.845	0.280	0.003		

ZBMI : body mass index z score

B: un-standardized regression coefficient

SE: standard error

*p*: *p*-value testing the significance of the regression coefficient

R^2^: variance explained by the regression model

*p* (△F): p-value testing the significance of F change from the preceding model (^#^ model 1 includes only the intercept term)

ref: reference group of the categorical variable that analyzed by creating dummy variables

*Cardiometabolic risk score **α** = Sum of components’ z score: Components of cardiometabolic risk score **α** include z-score of sex-specific, age-adjusted waist circumference, systolic and diastolic blood pressure (also height-adjusted), fasting plasma glucose, triglyceride and low-density lipoprotein cholesterol (LDL-C), and minus z-score of sex-specific age-adjusted high-density lipoprotein cholesterol (HDL-C)

The hierarchical regression results for the cardiometabolic summary risk score **β,** which included all components of risk score **α** except WC, were similar to the **α** score although a lower proportion of variance explained in each model (Table 3b). In Model 1, ZBMI could explain a significant proportion of the variance in the summary risk score **β** in both boys [R^2^ = 14.7 % (10.2 % - 19.2 %)] and girls [R^2^ = 6.6 % (3.8 % - 9.4 %)]. In Model 2, puberty further increased the variance explained significantly in both sexes [R^2^ = 15.5 % (10.9 % - 20.1 %) in boys and R^2^ = 7.9 % (4.9 % - 10.9 %) in girls]. When the interaction-term of ZBMI and puberty was included in Model 3, a further 0.8 % increase in the variance explained (*p* = 0.03) was found in boys [R^2^ = 16.3 % (11.7 % - 20.9 %]. For girls, there was a further 0.5 % increase in the variance explained [R^2^ = 8.4 % (5.3 % - 11.5 %)] but the change in R^2^ was not statistically significant (*p* = 0.051).

**Table 3b:** Effect of pubertal stage interaction on association of cardiometabolic risk score β by BMI

Cardiometabolic Risk Score β *	Boys (*n* = 828)					Girls (*n* = 1157)				
	B	SE	*P*	R^2^ (95 % CI)	*p* (△F)	B	SE	*p*	R^2^ (95 % CI)	*p* (△F)
Model 1				14.7 % (10.2 % – 19.2 %)	<0.001^#^				6.6 % (3.8 % – 9.4 %)	<0.001^#^
ZBMI	1.020	0.086	<0.001			0.619	0.069	<0.001		
Model 2				15.5 % (10.9 % – 20.1 %)	0.019				7.9 % (4.9 % – 10.9 %)	<0.001
ZBMI	1.076	0.089	<0.001			0.797	0.082	<0.001		
Pubertal stage:										
Pre-pubertal (ref)										
Pubertal	−0.415	0.226	0.067			−0.229	0.224	0.306		
Late/Post-pubertal	−0.647	0.230	0.005			−0.804	0.236	0.001		
Model 3				16.3 % (11.7 % – 20.9 %)	0.030				8.4 % (5.3 % – 11.5 %)	0.051
ZBMI	0.811	0.158	<0.001			1.328	0.239	<0.001		
Pubertal stage:										
Pre-pubertal (ref)										
Pubertal	−0.303	0.230	0.189			−0.801	0.324	0.014		
Late/Post-pubertal	−0.549	0.233	0.019			−1.314	0.316	<0.001		
Interaction terms:										
ZBMI * Pubertal	0.561	0.216	0.010			−0.675	0.283	0.017		
ZBMI * Late/Post-pubertal	0.192	0.222	0.389			−0.565	0.262	0.031		

ZBMI: Body mass index (BMI) z score

B: un-standardized regression coefficient

SE: standard error

*p*: *p*-value testing the significance of the regression coefficient

R^2^: variance explained by the regression model

*p* (△F): p-value testing the significance of F change from the preceding model (^#^model 1 includes only the intercept term)

ref: reference group of the categorical variable analyzed by creating dummy variables

* Cardiometabolic risk score β includes all components of risk score α except waist circumference

The above hierarchical regressions indicate that the effect of ZBMI on cardiometabolic risk scores **α** and **β** would depend on the pubertal stage. In particular, the marginal effect of increasing 1 unit of ZBMI on the cardiometabolic risk score **α** would be further increased by an average of 0.593 and 0.177 units to the score for boys in pubertal stage and late/post-pubertal stage respectively, as compared with boys in pre-pubertal stage (Table 3a). Such interaction effect of pubertal stage was, however, reversed in girls; the marginal effect of increasing 1 unit of ZBMI on the cardiometabolic risk score **α** was decreased by an average of 0.903 and 0.845 units for girls in pubertal stage and late/post-pubertal stage, respectively, as compared with girls in pre-pubertal stage (Table 3a). A similar pattern of the interaction effect of pubertal stage on the association between ZBMI and cardiometabolic risk scores **β** was observed (Table 3b).

## Discussion

Our study examined the association between puberty and CMRFs and the interaction effect of pubertal stage on the association of cardiometabolic risk by BMI from a cross sectional cohort of 1985 children aged 6–18 years. Among boys, puberty was significantly associated with some of the CMRFs, including increased WC, high TG levels, high BP, overweight and obesity, with the highest rate of these CMRFs among the pre-pubertal group. Among girls, puberty was significantly associated with increased WC and high BP, with the highest rate of these risk factors in late/post-pubertal and pre-pubertal groups, respectively. The pubertal stage was also found to have an interaction effect on the association of cardiometabolic risk by BMI. The models that included the interaction of BMI and puberty both had a significant increase in the proportion of the variance explained (Table 3a).

In our study population of Hong Kong Chinese children, we found a higher rate of CMRFs among pre-pubertal boys. This is a unique and interesting finding in that most studies have found an association between pubertal stage and CMRFs [22] including an increased WC [23], insulin resistance [24] and BP [25], as well as adverse lipid profiles [26]. In the present study, the higher rate of some CMRFs such as WC, TG, and BP for boys in their pre-pubertal stage may imply that the cutoffs of CMRFs have been set at a lower point for that stage and, therefore, have over-classified some boys as having CMRFs. Although there was no significant association found between pubertal stage and CMRFs clustering, this may be due to the small sample size of the cases. Further study warrants an investigation of the CMRFs cutoffs and the relationship between pubertal stage and CMRFs clustering for children.

One of the major issues at puberty is the difference in the percentage of body fat between sexes. Before puberty, both sexes have similar amounts of fat mass from age 5 until about 10 years. However during puberty girls in general experience an increase in the percentage of body fat while boys experience a decrease in the percentage of body fat [27]. Evidence suggests that the timing of pubertal development affects body composition in girls [28] and in boys [29, 30]. We have found a higher rate of increased WC among pre-pubertal boys with a decreasing trend towards late/post-puberty, but the reverse trend was found in girls. Consistent with our findings, studies have found that overweight and obese boys may also enter puberty later than thin boys [8, 11]. The difference that we observed between sexes in the association between WC and pubertal stage may be attributable to the physiological changes of body composition and hormones among pubertal females and males [10, 31]. As boys can retain a relative constant fat mass throughout pubertal development while gaining in height, this may explain the lower rate of increased WC and overweight and obesity among the late/post-pubertal boys. Likewise, it is possible that the higher rate of increased WC in the pre-pubertal boys may reflect the cross-sectional nature of the study and that over-nutrition, physical inactivity and sedentary lifestyle are particular problems in this young age group in our sample [25, 32]. The greater gain of fat mass, the hormonal status and normal physiological changes in body composition during puberty in girls may also account for the higher rate of increased WC among late/post-pubertal girls [8, 10].

Our results contrast with previous research on the association between puberty and BMI and various metabolic profiles [22, 33]. One possible reason for these differences could be our different analytical approach, but it is necessary to be cautious in making direct comparisons between our results and previous work. We explored the association between pubertal stages and individual CMRFs to examine whether the pubertal stage was associated with CMRFs clustering, whereas the other studies looked into the changes of metabolic profile that occur in different pubertal stages [22, 33]. This different approach may shed new light into the relationship between CMRFs and pubertal stage. We have shown an interaction effect of pubertal stage on the association of cardiometabolic risk by BMI in both boys and girls. Association of cardiometabolic risk score **β** is statistically more demanding than cardiometabolic risk score ***α*** as it removes the WC component from the risk score. Nevertheless, the increases in the variance explained by the interaction-term in both sexes were similar in both the scores ***α*** (ranging from 0.5 % to 0.7 %) and **β** (0.5 % - 0.8 %). These findings suggest that there may be an interaction effect of pubertal stage on the association of cardiometabolic risk by BMI. This means that the association of cardiometabolic risk by BMI depends on the stage of puberty. To our knowledge, few existing anthropometry references are able to account for children’s pubertal stage. One study has demonstrated that adjustment for sexual maturation can affect the estimates of overweight prevalence [34]. Further studies are warranted to assess how we can apply this information in clinical practice and to revisit the cutoffs for CMRFs which incorporate the pubertal stage of the child in the assessment.

### Limitations

Our study has a number of important limitations. First, we used self-reported Tanner stages to categorize the pubertal stages of the study participants. Although we have previously confirmed the utility of this self-reported Tanner pubertal questionnaire, self-reporting of puberty may still be difficult for children to determine their pubertal stage, particularly for stages 3 and 4 since sexual maturation stages are somewhat subjective and there is no exact cutoff of the Tanner stages [18]. Discrepancies between self-reported and the actual pubertal stage could exist and this is especially relevant in girls with obesity, and hence may affect the results. We reclassified the 5 Tanner pubertal stages into 3 groups: pre-pubertal, pubertal and post-pubertal which may help minimize under- or over-estimating the self-reported pubertal stage. Second, limitation of our analysis of the association between pubertal stage and CMRFs was the relatively small number of subjects who had a clustering of CMRFs. In addition, this study lacks sex hormone data owing to funding limitations. A future outcome trial with prospective data is suggested as it would address the mechanisms underlying the associations between puberty and CMRFs. Third, we did not assess the inter- or intra-observer reliabilities of the anthropometric measurements, although all the measurements were performed by experienced research staff who all had participated in collecting such data in school age children in our previous large-scale school children cohort study [32]. Furthermore, the multi-stage clustering sampling method used might introduce sampling bias to the results even though the schools and classes of students were randomly drawn in each corresponding stage of sampling from priori compiled sampling frames using data from the Hong Kong Education and Manpower Bureau. Last, this was a cross-sectional study and no moderation or causal relationships could be established even though there was an interaction effect of pubertal stage on the association between cardiometabolic risk and BMI. A longitudinal study is needed to further examine any moderation effect or causal relationship.

## Conclusions

We were able to show that CMRFs, including central obesity and high BP for both boys and girls, as well as high TG for boys, were associated with pubertal stage. Pubertal stage was found to have an interaction effect on the association of cardiovascular risk by BMI in boys and may have a potential interaction effect in girls. Further documentation of these findings in larger studies is required to determine how best to adjust for pubertal stage in studies related to obesity and CMRFs.

## Abbreviations

CMRFs: Cardiometabolic risk factors
HDL-C: High-density lipoprotein cholesterol
BMI: Body mass index
TC: Total serum cholesterol
WC: Waist circumference
LDL-C: Low-density lipoprotein cholesterol
BP: Blood pressure
TG: Triglyceride
DBP: Diastolic blood pressure
FPG: Fasting plasma glucose
BW: Body weight
SM: Early sexual maturation
BH: Body height
α: Alpha
B: Beta
